# Racial, Ethnic, and Geographic Differences in Vaginal Birth After Cesarean Delivery in the US, 2011-2021

**DOI:** 10.1001/jamanetworkopen.2024.12100

**Published:** 2024-05-17

**Authors:** Rana F. Chehab, Assiamira Ferrara, William A. Grobman, Mara B. Greenberg, Amanda L. Ngo, Emily Z. Wang, Yeyi Zhu

**Affiliations:** 1 Division of Research, Kaiser Permanente Northern California, Oakland; 2 Division of Maternal-Fetal Medicine, Department of Obstetrics and Gynecology, The Ohio State University College of Medicine, Columbus; 3 Department of Obstetrics and Gynecology, Kaiser Permanente Northern California, Oakland; 4 Regional Perinatal Service Center, Kaiser Permanente Northern California, Santa Clara; 5 Department of Epidemiology and Biostatistics, University of California, San Francisco

## Abstract

This cross-sectional study examines racial, ethnic, and geographic differences in vaginal birth after cesarean delivery in the US, from 2011 to 2021.

## Introduction

Cesarean delivery rates are higher in the US than in other high-income countries.^1^ Vaginal birth after cesarean (VBAC) is associated with reduced risk of maternal morbidity and future pregnancy complications vs cesarean delivery.^2^ The VBAC rate increased in the US from 10.7% in 2010 to 16.2% in 2020,^3^ but it is unknown whether racial, ethnic, and geographic differences exist. We assessed trends in VBAC rate by race, ethnicity, and geographic region in the US from 2011 to 2021.

## Methods

This was a repeated cross-sectional analysis of data, captured by the National Center for Health Statistics, of singleton deliveries in the US from 2011 to 2021 among individuals aged 15 to 44 years with a history of cesarean delivery. Race and ethnicity, recognized as social constructs, were self-reported by pregnant individuals. We classified states of birth into the 4 US Census Bureau regions.

We estimated age-standardized VBAC rate with 95% CIs and quantified trends from 2011 to 2021 using annual percentage change (APC) by Joinpoint Regression. Kaiser Foundation Research Institute’s institutional review board deemed this study exempt. Technical details are available in the eAppendix in Supplement 1.

## Results

Among 5 841 858 individuals, the mean (SD) age at delivery was 30.7 (5.3) years, with 57 487 (1.0%) American Indian or Alaska Native, 369 544 (6.3%) Asian or Pacific Islander, 926 332 (15.9%) Black, 1 496 958 (25.6%) Hispanic, 2 860 802 (49.0%) White, and 130 735 (2.2%) multiracial individuals. The overall age-standardized VBAC rate increased from 10.0% (95% CI, 9.9%-10.1%) in 2011 to 14.7% (95% CI, 14.6%-14.9%) in 2021 (APC, 3.9%; 95% CI, 3.3%-4.4%).

**Figure 1.**
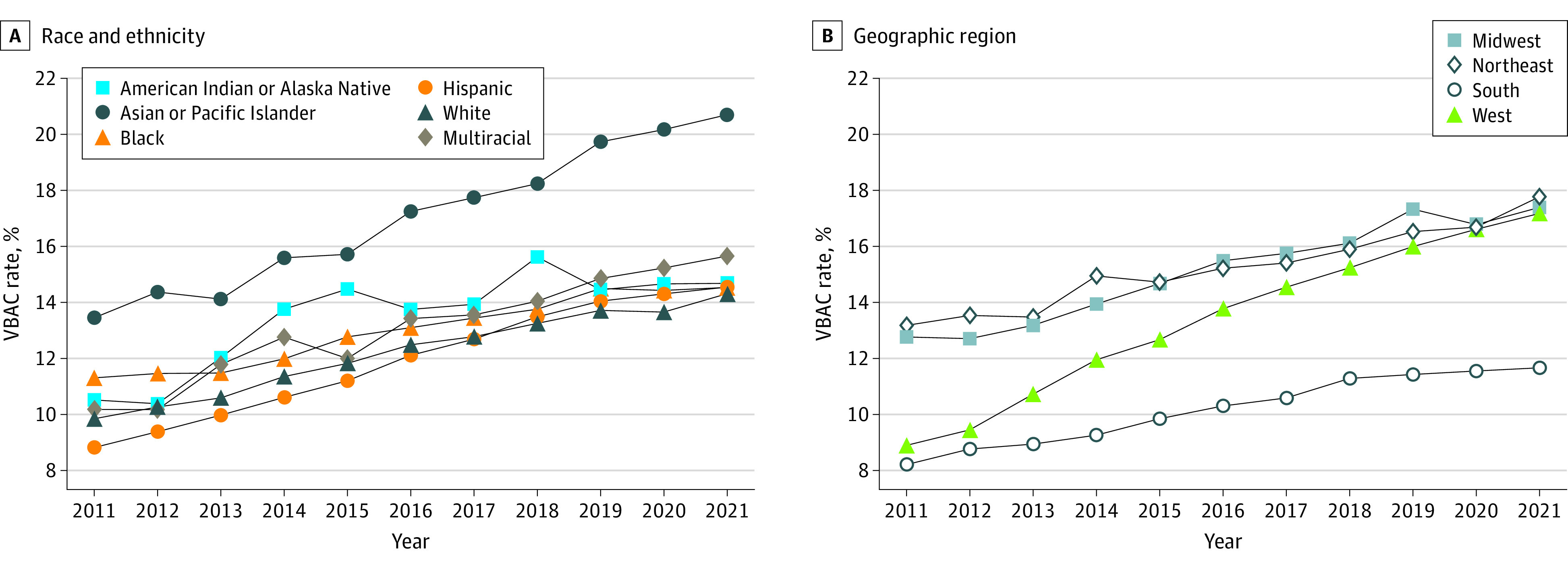
Trends in the Age-Standardized Rate of Vaginal Birth After Cesarean (VBAC) Among Individuals With Previous Cesarean Deliveries in the US, 2011-2021, by Race, Ethnicity, and Geographic Region A, The increase in VBAC rate by race and ethnicity was largest among Hispanic individuals (5.2%; 95% CI, 4.9%-5.5%), followed by Asian or Pacific Islander (4.7%; 95% CI, 4.1%-5.2%), multiracial (4.3%; 95% CI, 3.4%-5.2%), American Indian or Alaska Native (4.1%; 95% CI, 1.7%-6.7%), White (4.0%; 95% CI, 3.5%-4.5%), and Black (3.0%; 95% CI, 2.5%-3.4%) individuals. B, The largest increase in VBAC rate by geographic region was in the West (6.9%; 95% CI, 6.1% to 7.7%), followed by Midwest (3.6%; 95% CI, 3.0% to 4.2%), South (3.6%; 95% CI, 3.0% to 4.1%), and Northeast (2.9%; 95% CI, 2.4% to 3.3%

**Figure 2.**
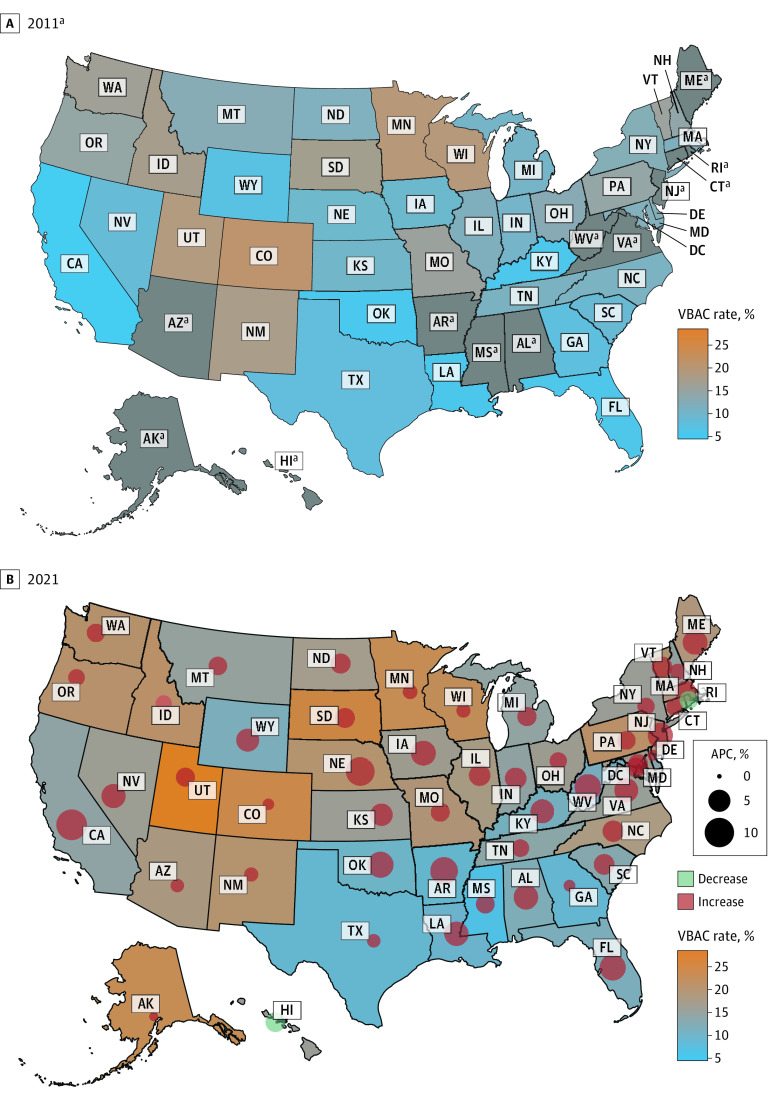
Trends in the Age-Standardized Rate of Vaginal Birth After Cesarean (VBAC) Among Individuals With Previous Cesarean Deliveries in the US, 2011-2021, by State of Birth APC indicates annual percentage change. ^a^Some states had not adopted the 2003 revised version of the birth certificate by 2011. The trend in these states was examined from the first year they adopted the 2003 revised version, which is as follows: 2012, Virginia; 2013, Alabama, Maine, and Mississippi; 2014, Alabama, Arizona, Arkansas, Hawaii, and West Virginia; 2015, Rhode Island; and 2016, Connecticut and New Jersey. All other states had adopted the revised version by 2011.

Trends by race and ethnicity are shown in Figure 1A. Trends by geographic region are shown in Figure 1B. Most states had increases in VBAC rate, with the largest increase in California (11.1%; 95% CI, 10.0% to 12.2%), whereas 2 states, Hawaii (−3.6%; 95% CI, −7.1% to 0.1%) and Rhode Island (−2.8%; 95% CI, −8.6% to 3.4%), had decreases, although the results were not statistically significant (Figure 2).

## Discussion

Using US nationally representative data, in this cross-sectional study we report a nearly 50% increase in VBAC rate from 2011 to 2021, with lingering racial, ethnic, and geographic differences in absolute rate and APC. Black individuals had the smallest and Hispanic individuals had the largest increase in VBAC rate from 2011 to 2021, with both groups having lower VBAC rates than the overall population in recent years. It is critical to monitor race- and ethnicity-specific VBAC rates to expose maternal health inequities while acknowledging that race and ethnicity are not risk factors per se but proxies for root causes of health disparities such as structural racism.^4^

The South maintained the lowest VBAC rate from 2011 to 2021 despite having an APC similar to the Midwest. The West, on the other hand, had a VBAC rate similar to the South in 2011 but had the largest APC and reached a rate similar to the Midwest and Northeast in 2021. Geographic differences in VBAC rate may be associated with contextual factors, including health care access and availability and training of obstetric care practitioners.^5^

Study limitations include that VBAC rate calculations before 2016 might be affected by the exclusion of birth certificates that did not use the 2003 revised version; nevertheless, a sensitivity analysis of deliveries from 2016 to 2021 yielded results similar to the main analysis. Furthermore, misclassification in birth certificate reporting is possible, but mode of delivery in the birth certificates has been previously validated.^6^

Our findings reflect lingering racial, ethnic, and geographic differences in VBAC rates, highlighting the need for person-centered clinical counseling and management of pregnant individuals with a history of cesarean delivery and public health efforts to promote health equity. Future research is warranted to better understand the root causes of these differences to inform targeted strategies toward more equitable maternal health care.

## Notes

Supplement 1. **eAppendix. **Supplemental Methods
eReferences

Supplement 2.
Data Sharing Statement
