# Supplementary material for: Racial, Ethnic, and Geographic Differences in Vaginal Birth After Cesarean Delivery in the US, 2011-2021
Source: JAMA Netw Open. 2024 May 17;7(5):e2412100. doi: 10.1001/jamanetworkopen.2024.12100 (PMC11102014; doi:10.1001/jamanetworkopen.2024.12100)
Supplement: Supplement 1. — eAppendix. Supplemental Methods eReferences [file jamanetwopen-e2412100-s001.pdf]

Supplemental Online Content

Chehab RF, Ferrara A, Grobman WA, et al. Racial, ethnic, and geographic differences in vaginal birth after cesarean delivery in the US, 2011-2021. *JAMA Netw Open*. 2024;7(5):e2412100. doi:10.1001/jamanetworkopen.2024.12100

eAppendix. Supplemental Methods

eReferences

This supplemental material has been provided by the authors to give readers additional information about their work.

© 2024 Chehab RF et al. *JAMA Network Open.*

eAppendix. Supplemental Methods

Data source and variables:

We reviewed birth certificates from the National Center for Health Statistics (NCHS) that captured all live births in the US between 2011-2021. The attendant clinician at birth completes the birth certificates from pregnant individual's self-report and medical records based on a standardized protocol by NCHS.[1]

Race and ethnicity, which are recognized as social constructs rather than biological determinants, were self-reported by pregnant individuals,[2] and categorized as follows: American Indian or Alaska Native (AIAN), non-Hispanic Asian/Pacific Islander (referred to as Asian/PI), non-Hispanic Black (Black), Hispanic/Latina (Hispanic), and non-Hispanic White (White). We categorized AIAN individuals irrespective of Hispanic ethnicity based on the Centers for Disease Control and Prevention's recommendations,[3] and we used single-race groups with an additional category of multiracial since bridged race groups were no longer reported in birth certificates starting in 2020.[2] Age at delivery (years) was self-reported by the pregnant individual. Previous cesarean delivery (yes/no) and mode of delivery of the index pregnancy (vaginal/cesarean) were extracted from medical records.

Year of birth and state of birth, categorized into four regions (Northeast, Midwest, South, and West) based on the US Census Bureau regions,[4] were reported in the birth certificate.

There was no missing information on race, age at delivery, year of birth, and state of birth. A small number of birth certificates had missing information on ethnicity (0.83%), previous cesarean delivery (0.16%), and mode of delivery of the index pregnancy (0.07%). The socio-demographic characteristics of individuals with complete vs. incomplete data are similar.

Exclusion criteria:

We excluded birth certificates not using the 2003 revised version because previous versions reported the mode of delivery using different non-comparable format and wording.[5] In addition, we excluded birth certificates of individuals younger than 15 years or older than 44 years, non-US residents, and individuals with nonsingleton births to avoid duplication of maternal data because certificates could not be linked in the deidentified data set. We also excluded birth certificates of individuals with a history of no previous cesarean delivery and those with missing data on ethnicity or mode of delivery.

Statistical analysis:

We age-standardized VBAC rates to the age distribution of US individuals who gave birth in 2011, the beginning of the study period. We estimated age-standardized VBAC rate and 95% CI per 100 live births overall and by race and ethnicity, geographic region, and state of birth.

To quantify trends in VBAC rate over time, we used Joinpoint Regression version 4.7.0.0 to calculate mean annual percent change (APC).[6] Joinpoint Regression accounts for potential nonlinear trends by allowing for identification of an inflection point. Where an inflection was identified indicating a nonlinear trend, mean annual change was calculated based on the weight

© 2024 Chehab RF et al. *JAMA Network Open.*

of the component trend segments. If no inflection point was identified, mean APC indicates the annual change in VBAC rate across a linear trend.

Some states had not adopted the 2003 revised version of the birth certificate by 2011, so we included these states in the trend analysis at the first year when they adopted the 2003 revised version. For example, Rhode Island adopted the 2003 revised version in 2015, so we examined the trend of VBAC rate in Rhode Island from 2015-2021. The first year after 2011 when these states adopted the 2003 revised version of the birth certificate is as follows: 2012, Virginia; 2013, Alaska, Maine, and Mississippi; 2014, Alabama, Arizona, Arkansas, Hawaii, and West Virginia; 2015, Rhode Island; and 2016, Connecticut and New Jersey. All other states had adopted the 2003 revised version by 2011.

© 2024 Chehab RF et al. *JAMA Network Open.*

eReferences

1. National Center for Health Statistics. Guide to completing the facility worksheets for the Certificate of Live Birth and Report of Fetal Death. *Hyattsville, MD: National Center for Health Statistics*. 2016;
2. Centers for Disease Control and Prevention. *User Guide to the 2020 Natality Public Use File*. 2020. Accessed January 2023.
3. Centers for Disease Control and Prevention. Office of Management and Budget (OMB) DIRECTIVE NO. 15 Race and Ethnic Standards for Federal Statistics and Administrative Reporting. Accessed 10/2/2022, https://wonder.cdc.gov/wonder/help/populations/bridged-race/directive15.html
4. United States Census Bureau. Geographic Levels. https://www.census.gov/programs-surveys/economic-census/guidance-geographies/levels.html
5. MacDorman M, Declercq E, Menacker F. Recent trends and patterns in cesarean and vaginal birth after cesarean (VBAC) deliveries in the United States. *Clin Perinatol*. Jun 2011;38(2):179-92. doi:10.1016/j.clp.2011.03.007
6. National Cancer Institute. Joinpoint Trend Analysis Software Version 4.9.1.0. https://surveillance.cancer.gov/joinpoint/

© 2024 Chehab RF et al. *JAMA Network Open.*
