# Supplementary material for: Racial, Ethnic, and Geographic Differences in Vaginal Birth After Cesarean Delivery in the US, 2011-2021
Source: JAMA Netw Open. 2024 May 17;7(5):e2412100. doi: 10.1001/jamanetworkopen.2024.12100 (PMC11102014; doi:10.1001/jamanetworkopen.2024.12100)
Supplement: Supplement 2. — Data Sharing Statement [file jamanetwopen-e2412100-s002.pdf]

# Data Sharing Statement

Chehab. Racial, Ethnic, and Geographic Differences in Vaginal Birth After Cesarean Delivery in the US, 2011-2021. *JAMA Netw Open*. Published May 20, 2024.
doi:10.1001/jamanetworkopen.2024.12100

**Data**
**Data available:** Yes
**Data types:** Deidentified participant data
**How to access data:** Data are deidentified and publicly available in the Vital Statistics Online Data Portal accessible at: https://www.cdc.gov/nchs/data_access/vitalstatsonline.htm
**When available:** With publication

**Supporting Documents**
**Document types:** None

**Additional Information**
**Who can access the data:** All data are publicly available to all researchers.
**Types of analyses:** For any purpose.
**Mechanisms of data availability:** All data are publicly available to all researchers.
**Any additional restrictions:** N/A.
